# Physico-Chemical and Microstructural Changes during the Drying of Persimmon Fruit cv. Rojo Brillante Harvested in Two Maturity Stages

**DOI:** 10.3390/foods9070870

**Published:** 2020-07-03

**Authors:** Nariane Q. Vilhena, Rebeca Gil, Empar Llorca, Gemma Moraga, Alejandra Salvador

**Affiliations:** 1 Postharvest Department, Instituto Valenciano de Investigaciones Agrarias, 46113 Valencia, Spain; quaresma_nar@gva.es (N.Q.V.); gil_reb@gva.es (R.G.); 2 Food Technology Department, Universitat Politècnica de València, 46022 Valencia, Spain; emllomar@tal.upv.es (E.L.); gemmoba1@tal.upv.es (G.M.)

**Keywords:** *Diospyros kaki* Thunb., cryo-FESEM, semidried, air drying, quality characteristics

## Abstract

The physico-chemical and microstructural changes of “Rojo Brillante” persimmons in two maturity stages (S1 and S2) were evaluated during air drying. The maturity stage influences moisture loss. A Moisture level of approximately 50%, a limit at which persimmons are considered semidried, was reached after 21 and 28 days for S1 and S2, respectively. Shrinkage resulting from water removal led to secondary epidermis formation concomitantly to internal flesh gelling, which was related to moisture loss and water activity changes of each fruit part. The thicker epidermis and the lower volume of gelled area inside the S1 fruits led to harder fruit compared to the S2 fruits. The microstructural study revealed parenchyma degradation during drying in both the outermost area (secondary epidermis) and internal flesh, and this process was faster in S1 than in S2. The second peel presented hollows, generated by water outflow, which were bigger in S1 and explained the faster internal dehydration in S1. During drying, slight browning occurred, as reflected in the declining color parameters (L*, h* and C*). Water removal led to soluble solids tannin reduction to non-astringency values on day 28.

## 1. Introduction

Persimmon fruit (*Diospyros kaki* Thunb.) is a species that originates from China and was introduced to Europe in the 17th century [1]. Spain recently became the second biggest producer of persimmon fruit in the world, with an expected production of 600,000 tons by 2020 centered mainly around the astringent cultivar “Rojo Brillante” due to its high quality and adaptation to climate conditions [2,3].

In Europe, the marketing season of persimmon goes from November to January, in late autumn and early winter, with a plentiful supply. However, the accelerated maturation process of persimmon reduces its availability all year round and increases the number of post-harvest losses, which can be as high as 20% of production [4,5]. In this context, the productive sector faces the challenge of introducing new product options to reduce losses associated with persimmon fruit management and seasonality.

The drying process is a usual and simple technique of which its aim is fruit preservation to extend the period that it is available. The production and consumption of semidried persimmons are traditional in Asian countries like South Korea, China and Japan, with a variety of processing methods to generate a new high quality and stable product, with good sensory attributes [6,7].

Dried persimmon can be classified into dried and semidried based on its water content. Although the moisture limit for this classification varies between countries and varieties, on average semidried persimmons are processed at around the 50% moisture level, whereas dried fruits can reach about 35% [8,9].

The production of dried persimmons enables the commercialization and exportation period to be prolonged as reduced moisture content and consequently, volume and weight improve shelf life due to low microbial and biochemical degradation, which reduces packaging, storage and transportation costs [10]. Drying also emerges as a management option because this process also reduces astringency in some astringent varieties. In China, this is the main way to commercialize astringent persimmons, which are more suitable for drying than non-astringent cultivars because they turn brown and tough [7,11].

Despite being a consolidated technique in several countries and the fact a considerable number of studies have been published about drying methods, changes in quality characteristics, and nutritional benefits in these regions, in Spain, there is still no record of semidried persimmon production or published references about the effects of the drying process on fruit. Therefore, this study aims to provide information on the physico-chemical and microstructural changes that occur during drying “Rojo Brillante” persimmon fruit harvested in two maturity stages.

## 2. Materials and Methods

### 2.1. Fruit Samples and Experimental Procedure

Persimmons fruit cv. Rojo Brillante were harvested from commercial orchards at l’Alcudia (Valencia, Spain) in two maturation stages. The criteria for harvesting each maturity stage were firmness and external color index. In persimmon, the external color is the most common non-destructive index for harvesting and a strong negative correlation between skin color and firmness values during maturation has previously been reported [12,13]. *L*, *a*, *b* Hunter parameters were measured and results were expressed as a skin color index as in Equation (1):

(1) $$CI=1000\times \frac{a}{L\times b}$$

where *CI* is the color index and *L*, *a* and *b* are the Hunter Lab parameters of color scale.

The early stage (S1) had firmness values of 49.3 N ± 6.9 and a peel color index of 6.7 ± 2.0 and the late stage (S2) had firmness values of 31.5 N ± 5.8 and a peel color index of 21.6 ± 2.1. Harvests took place throughout November. 

After harvests, fruit were transported to the Instituto Valenciano de Investigaciones Agrarias (IVIA; Moncada, Spain) where they were selected according to homogenous color and absence of external damage. Then 120 fruits from each maturity stage were peeled off manually with a peeler to be subsequently immersed for 10 min in 4.5% sodium metabilsufite (Na_2_S_2_O_5_) solution, which is used as a disinfectant, antioxidant, and preservative agent [11].

The fruit were placed on hooks and hung by the pedicel for natural drying in the IVIA’s pilot plant for up to 63 days. Six fruits were placed on each hook to separate stages 1 and 2. Average temperature and relative humidity during the drying period were taken from the IVIA weather station and ranged from 6 °C to 17 °C and 49% to 83%, respectively.

### 2.2. Determinations

The initial fruit characterization was made with 20 fruits from each maturity stage. The same analyses were carried out once weekly with 12 fruit (two hooks) from each stage. Three pieces of fruit were used for the moisture and water activity determinations and the microstructural study. Nine were employed for the other physico-chemical analyses. All the analyses were determined based on individual fruits.

#### 2.2.1. Weight and Diameters

Fruit weight was individually measured on a precision scale (model PB3002-S/FACT, Mettler Toledo, Switzerland). The longitudinal and equatorial diameters, as well as the thickness of the generated secondary epidermis due to the drying process, were measured by a pachymeter (Mitutoyo 500-267V-CDL20CP, Japan).

#### 2.2.2. Moisture and Water Activity

The fruit were cut into two parts. One half was used to measure the moisture and water activity of the whole fruit. In the other half, the outmost area (secondary epidermis) was carefully separated with a scalpel blade from the rest of the pulp (internal flesh) in order to measure the moisture and water activity in each of these parts. 

Samples were ground in a crushing machine, of which its water content (x_w_) and water activity (a_w_) were measured by a Vaciotem, J.P. Selecta vacuum oven (60 ± 1 °C, pressure <100 mm Hg) and an Aqualab CX-2 Decagon Device, respectively. Three replicates were measured per fruit.

#### 2.2.3. Firmness and Color

Firmness was measured in a Texturometer Instron Universal Machine (model 4301, Instron Corp., Canton, MA, USA) using a 35 mm flat plunger. It approached fruit in such a way that subjected it to a compression force of 10 N along its equatorial axis. The relation between the deformation produced by the applied force and the initial fruit diameter was expressed as millimeters of deformation.

Fruit color was evaluated after peeling with a Minolta Colorimeter (model CR-400, Ramsey, NY, USA). The lightness (L*), chroma (C*), and hue angle (h*) values of persimmons were measured directly on two opposite equatorial zones of each fruit.

#### 2.2.4. Total Soluble Solids and Tannin Content

These analyses were also carried out with whole fruits and by also separating the secondary epidermis (generated during drying) and internal fruit flesh. To determine total soluble solids (TSS), the samples of each fruit were crushed by a Polytron homogenizer (model PT 3100D, Kinematica, Switzerland). To avoid tannins interfering with total soluble solid measurements, the insolubilization of tannins was previously done following the method of Sugiura et al. [14]. Measurements were recorded with a refractometer (Atagomod. PR1) and the results were expressed as °Brix.

Soluble tannins (ST) were determined by the Folin-Denis method [15], as described by Arnal and Del Río [16], and the results were expressed as a percentage of dry weight (DW).

#### 2.2.5. Microstructure Analysis

The microstructural study was performed by Cryo-field emission scanning electron microscopy Ultra 55 FESEM (ZEISS, Oberkochen, Germany) (Cryo-FESEM). Cubes (3 mm^3^) were cut from the equatorial area perpendicularly to the main axis of the persimmon flesh with a stainless-steel cutter. These cubes were then immersed into slush nitrogen (−210 °C) and transferred to a cryo-trans GeminiSEM 500 (ZEISS, Oberkochen, Germany) linked with a field emission scanning electron microscope, which operated at a temperature below −130 °C. Samples were cryofractured at −180 °C and etched at −90 °C.

For the light microscopic (LM) analysis, issue sections were taken from the internal flesh using a stainless blade. Sections were placed on histological slides and stained with calcofluor (0.1%), a specific agent that identifies cell walls. Cell walls react with calcofluor and give a blue color [17]. Dyes were detected using a mercury arc lamp as the light source. The excitation and emission filters for calcofluor were 370/36–25 nm and 440/40–25 nm, respectively. A Nikon ECLIPSE 80i (Nikon Co., Ltd., Tokyo, Japan) light microscope was used. Samples were observed at the 4× magnification. Images were captured and stored at 1280 × 1024 pixels using the microscope software (NIS-Elements F, version 4.2, Nikon, Tokyo, Japan).

#### 2.2.6. Statistical Analysis

Data were subjected to analyses of variance (ANOVA) and multiple comparisons between means at *p* ≤ 0.05, determined by the LSD test. The Statgraphics Centurion XVII.I software application was used for the statistical analysis (Manugistics Inc., Rockville, MD, USA).

## 3. Results and Discussion

### 3.1. Weight Loss, Changes in Fruit Shape, Shrinkage and Secondary Epidermis Formation

**Figure 1:**
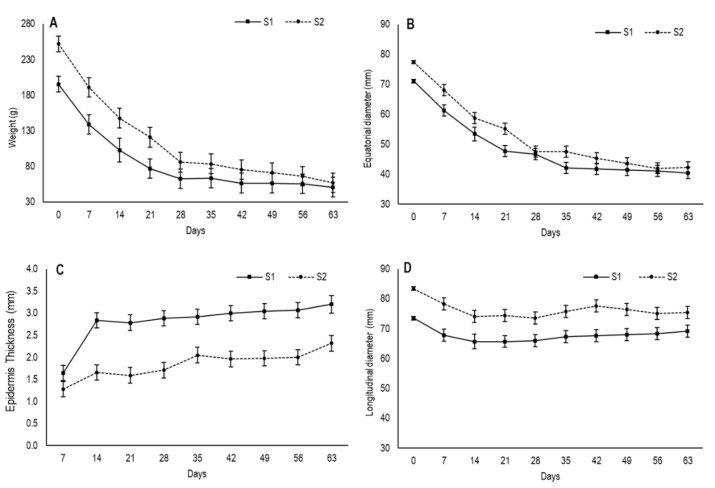
Changes in (**A**) weight, (**B**) equatorial diameter, (**C**) secondary epidermis thickness and (**D**) longitudinal diameter during the drying process of persimmon cv. Rojo Brillante in two maturity stages (S1 and S2). Vertical bars represent Least Significant Differences (LSD) intervals (*p* ≤ 0.05).

The drying process of foods reduces the total weight as water is removed from the material, which provides an overall volume reduction of whole fruit [18]. In the present study, the fruit in the first maturity stage (S1) had an initial weight of 195.3 g, which was lower than the fruit in the second one (S2), which came close to 252 g (Figure 1A). During the drying process, the fruit from both stages underwent considerable weight loss until approximately day 28. Then, while fruit weight remained stable for the S1 fruit, it continued to slowly decrease in the S2 fruit, with no significant differences over time. When drying ended, the fruit from both stages had an average weight of 54 g.

Fruit size changes were reflected by the values of longitudinal and equatorial diameter, which were measured during the drying process (Figure 1B,D). Concomitantly to the reduced fruit weight, the equatorial diameter descended gradually until day 28 in both stages and then showed a tendency to stabilize by reaching values of 40.4 cm and 42.3 cm for S1 and S2, respectively, at the end of the experiment, with no significant differences.

The longitudinal diameter showed slight variation throughout drying and varied from 73.5 cm to 69.2 cm for S1 and from 83.4 cm to 75.4 cm for S2 (Figure 1D). In both stages, the most marked decrease took place on the first 14 days, after which time no changes were observed. The slight variation observed in the longitudinal diameter during the drying process was probably due to the position at which fruit was hung by suffering the influence of gravity.

In this study, S2 had higher initial values for weight and diameters and displayed more weight loss than S1, probably due its larger evaporation surface. According to May and Perré [19], the surface area of drying products directly influences the drying rate, reporting that the longer the exchange surface of the fruit, the greater the water loss. These results are different to what Cho et al. [20] observed, who found that smaller sized fruits showed greater weight loss compared to bigger ones during the drying process.

**Figure 2:**
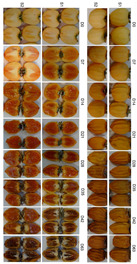
Captured images of persimmon cv. Rojo Brillante in two maturity stages (S1 and S2) during the drying process.

The reduction in the overall fruit volume during drying is usually defined as shrinkage [18]. Dry fruit shrinkage is not always uniform in the material dimension and depends on how uniformly water removal occurs in it. In the present study, shrinkage was accompanied by the outermost fruit zone evidently warping and wrinkling, which became more evident with drying time (Figure 2). This feature is caused by loss of internal volume due to water evaporation, which induces a moisture gradient and fruit display this distinctive quality [21,22].

During the drying process, shrinkage is usually concomitant to the formation of a rigid external layer that results from a high water evaporation rate from the external fruit zone, which loses moisture faster than the inner zone. This process is known as “Case Hardening” or “Crust Formation” and is either desirable or not in dried food products. In extreme cases, this process can lead to skin that is practically impervious to moisture, which encloses the volume of the material so that no interior moisture can be removed [18,21,23]. In persimmon fruit, Kang et al. [9] referred to this external rigid structure as a second peel or a secondary epidermis.

In this study, the second peel was already visible on day 7 for S1 and its thickness was 1.64 mm (Figure 1C). After 14 days, drastic thickening had occurred before stabilizing until day 35 and increasing again after day 40. The secondary epidermis had thickness values of 3.2 mm by the end of the assay. In S2, this layer was evident on day 14 and its thickness was 1.65 mm. Afterwards, it underwent no further changes until day 28, when it slightly increased, with values of 2.3 mm on day 63.

As the images reflect (Figure 2), the formation of this second peel was accompanied by a drastic change in the internal fruit structure. Internal flesh appears more gelatinous with the drying progress as the secondary epidermis causes resistance near the fruit surface, which fixes the volume inside the fruit and hinders water loss from the innermost region, which becomes softer and looks rubbery or has a gelatinous aspect [22]. In S1 after 7 days, the internal flesh texture drastically changed and displayed gelation symptoms. In S2, this flesh gelation started later and was visible after 14 days.

### 3.2. Changes in Moisture and Water Activity

**Figure 3:**
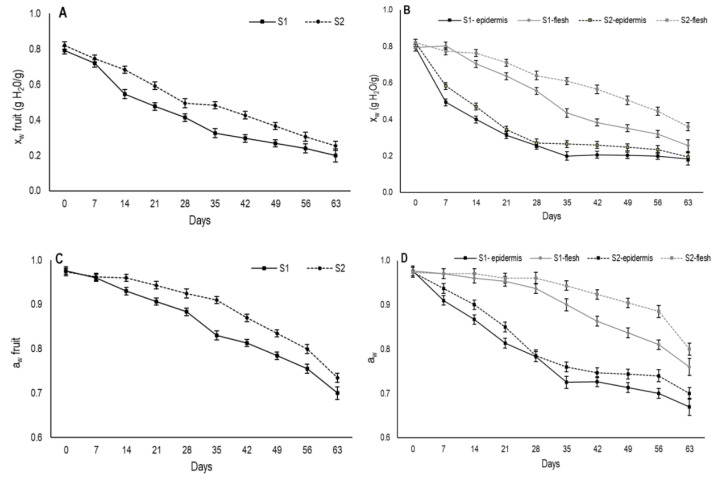
Changes in (**A**,**B**) moisture (x_w_) and (**C**,**D**) water activity (a_w_) of whole fruit and by parts (secondary epidermis and internal flesh) during the drying process of persimmon cv. Rojo Brillante in two maturity stages (S1 and S2). Vertical bars represent Least Significant Differences (LSD) intervals (*p* ≤ 0.05).

In the present study, during the drying period both moisture content and water activity were measured for the whole fruit (Figure 3A,C). Moreover, these parameters were also determined separately in the outer (secondary epidermis) and inner areas (internal flesh) of fruit from day 7 onward (Figure 3B,D). At harvest, moisture content came close to 0.8 gH_2_O/g for the fruit from both maturity stages and steadily lowered over the 63 days that the evaluation lasted (Figure 3A). The decline in moisture was faster in S1 fruit than in S2.

According to Kang et al. [9], moisture at around 50%, or 0.5 gH_2_O/g, is the limit at which persimmon fruits are traditionally named semidried. For the whole fruit, this value was reached on days 21 and 28 for S1 and S2, respectively. In the Asian market, semidried persimmons usually have a better market value for being characterized by a softer texture, which are more appealing compared to fully-dried fruit [24].

As expected, moisture loss in both maturity stages was significantly higher in the outer area than in the internal flesh (Figure 3B). In the outermost fruit area, moisture content sharply dropped during the first drying week for the fruits from both stages and this drop was more marked in S1. Afterward, moisture gradually lowered to days 35 and 28 for S1 and S2, respectively, from which time the moisture tended to stabilize and reach values that came close to 0.18 gH_2_O/g in S1 and 0.19 gH_2_O/g in S2.

The marked reduction in moisture in the outermost area during the first experimental weeks would be the cause of the second peel formation in the fruit. The faster external moisture loss exhibited in S1 fruit would explain the earlier formation of the second peel compared to fruit from S2. Moreover, the lower moisture content on the S1 fruit surface during the drying period induced more shrinkage in these fruits. It was noteworthy that when second peel formation was observed on day 7 and day 14 in S1 and S2, respectively, the moisture content of this area was similar and came close to 0.48 gH_2_O/g.

Moreover, in S1, this structure was thicker throughout drying, which would also be related to the more marked moisture loss in S1 compared to S2. According to Achanta and Okos [21], if the drying rate is slow enough that moisture lost from the product surface is replenished by moisture from the inside, crust formation may be inhibited. In this research, the slower drying rate in S2 produced a crust, but it was considerably thinner than S1.

Regarding changes in internal flesh moisture during drying, major differences were observed between both maturity stages (Figure 3B). S1 showed stable internal moisture until day 7, then it began to gradually decrease. From 28 days onwards, the internal moisture dropped more sharply until reaching values of 0.26 gH_2_O/g on day 63. In S2, a gradual moisture reduction took place from day 14, achieving a moisture value of 0.36 gH_2_O/g on day 63. Throughout drying, this stage showed higher moisture content than S1.

When comparing the weight loss dynamics to moisture variation in fruit, it is noteworthy that the most pronounced weight reduction took place during the first 28 days (Figure 1A), then the stabilization tendency followed the same pattern as the outer area moisture loss (Figure 3B). The moisture content in this area remained stable from 28 days onward, while internal flesh, with a higher moisture content, continued to slowly lose moisture. This behavior would result from the reduction in water evaporation and secondary epidermis hardening according to Rahman [18]. In addition, although moisture continued to constantly drop in the inner fruit area until 63 drying days in both stages, weight loss was not statistically significant because only a small amount of water continued to be lost.

Water activity is the relation between the vapor pressure of food in balance with the surrounding atmosphere and the vapor pressure of water under the same conditions. It represents the efficiency at which the water present in fruit can participate in chemical reactions [25,26]. Initial water activity values were measured and came close to 0.80 for the whole fruit in both stages (Figure 3C). A slight decrease was recorded until day 28 and day 35 in S1 and S2, respectively. From this time, a major decrease took place. Throughout the drying period, the aw values were always higher in S2 than in S1.

When aw was measured in the outermost area, a similar pattern to moisture was observed during the drying period (Figure 3D). A major reduction in aw was recorded until day 35 and day 28 for S1 and S2, respectively, when it slowed down. Nevertheless, in the internal flesh, aw did not change until day 21 for S1 and until day 28 for S2. After these dates, a gradual decrease was observed in both stages and the aw values were always higher for S2.

The formation of the secondary epidermis could influence the reduction in water activity in the internal flesh. In dried foods it has been reported that the crust formation may maintain high values of water activity in the flesh since it forms a hard layer containing the free water inside [27].

### 3.3. Changes in Firmness and External Color

The mechanical properties of dry fruits, such as firmness, can be related to the effect of water sorption. Dry fruit generally become softer due to the plasticizing effect of water, which leads to a depression in viscosity and loss of the crunchy/crispy texture [23]. However, depending on the drying type or intensity, hardness tends to increase again due to material shrinkage, which significantly compacts the structure, or due to excessive water evaporation and consequently, a higher concentration of solids [9,23].

**Figure 4:**
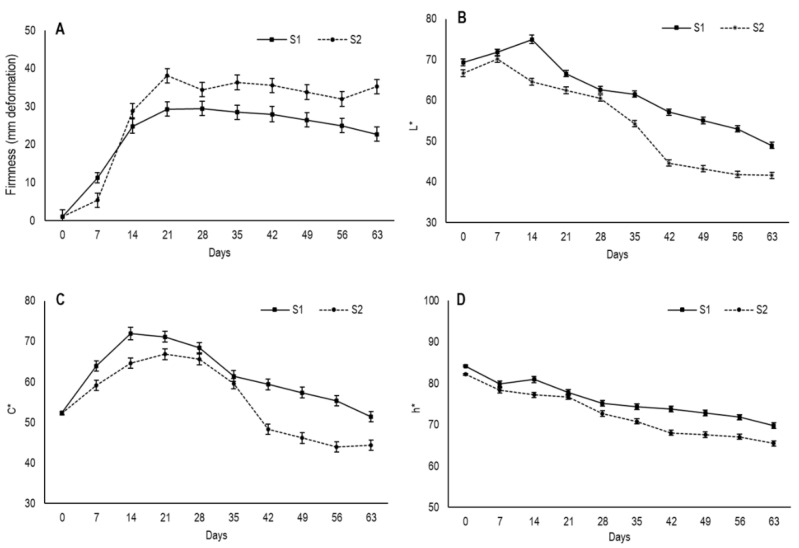
Changes in the (**A**) firmness, (**B**) lightness-L*, (**C**) chroma-C* and (**D**) hue angle-h* values during the drying process of persimmon cv. Rojo Brillante in two maturity stages (S1 and S2). Vertical bars represent Least Significant Differences (LSD) intervals (*p* ≤ 0.05).

The results showed that the lowest deformation value of flesh fruit was at harvest, when fruit were still completely firm (Figure 4A). From day 7, fruit underwent drastic softening until day 14 for S1 and until day 21 for S2, when firmness tended to stabilize with the deformation values of S2 fruits, which were always higher. This tendency to firmness stabilization was possibly due to moisture being maintained in the secondary epidermis during approximately the same period. From day 42, the deformation values of S1 slightly lowered, which indicates pulp hardening with an average deformation of 22.7 mm on day 63, while the average for S2 was 35.6 mm.

The lower deformation values in S1 compared to S2 could be due to the fruit in the first maturity stage developing a thicker secondary epidermis, which would imply greater resistance to deformation. Moreover, the smaller S1 fruit size would allow them to have a smaller gelatinous inner area volume, which would lead to less elasticity and more hardening.

Color is one of the most important sensory resources for determining consumer acceptability and product quality of dried food [28]. During persimmon drying, browning occurs which leads to evident fruit color changes [24,29]. The main factors reported to be involved in browning are the oxidative and nonoxidative degradation pathways of ascorbic acid [24]. Sodium metabisulfite solution, often used prior to drying to minimize microbial attacks, also prevents color deterioration during dehydration by retarding both enzymatic and nonenzymatic browning reactions [30,31]. In the present study, fruit were treated with sodium metabisulfite prior to drying, which proved effective in controlling pathogen attack. However, external fruit darkening took place.

To assess fruit external color, the values of the parameters lightness (L*), chroma (C*) and hue angle (h*) were recorded and are shown in Figure 4B–D. The maturity stage influences the evolution of these color parameters during the drying process. The L* values at harvest were 69.3 for S1 and 66.6 for S2, with a slight increase on the first 14 drying days for S1 and on the first 7 days for S2. Then in both cases, the L* values gradually lowered throughout what was left of the drying period, and the values in S1 were higher than S2. After 63 days, fruit had values of 48.9 and 41.6 for S1 and S2, respectively. In relation to C*, which measures color saturation, the fruit from both stages started with similar values, which came close to 52 and displayed increased saturation during the first few weeks, before lowering from day 21 in S1 and from day 28 in S2. By day 63, their average values were lower than their initial values, with 51.3 in S1 and 44.4 in S2.

Browning in semidried persimmon has been related to fruit water content. In “Atago” persimmon, browning has been reported to occur when fruit goes from semidried to dried, with water content close to 50% or less [24]. Accordingly, in the present study, the marked reduction in C* after 21 days in S1 and 28 days in S2 coincided with the time at which whole fruit moisture was about 50%, or 0.5 gH_2_O/g. This occurred when secondary peel moisture stabilized after a marked reduction.

Hue angle (h*) is related to color perception and indicates the primary color associated with angles of 0° (red), 90° (yellow), 180° (green) and 270° (blue) [32]. At harvest fruit had h* values of 84.2 and 82.2 for S1 and S2, respectively (Figure 4D). The Hue angle showed a gradual decrease in both maturity stages throughout drying and S1 fruits obtained higher values during the entire period. By day 63, h* values were 69.8 and 65.5 for S1 and S2, respectively.

### 3.4. Changes in Total Soluble Solids and Soluble Tannins

**Figure 5:**
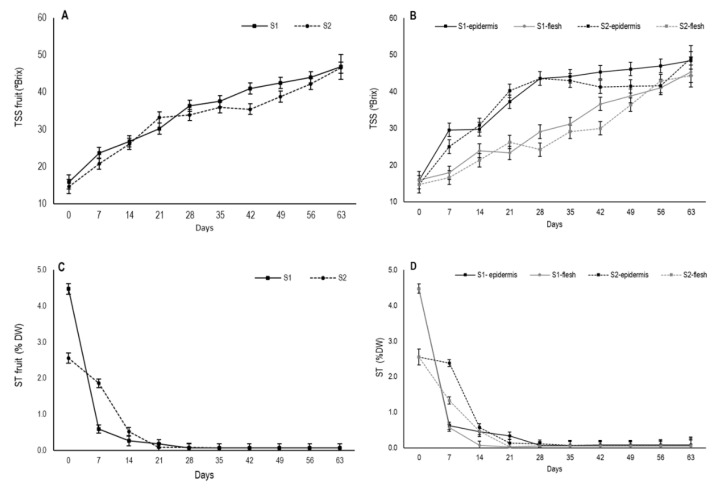
Changes in (**A**,**B**) total soluble solids (TSS) and (**C**,**D**) soluble tannins (ST) of whole fruit and by parts (secondary epidermis and internal flesh) during the drying process of persimmon cv. Rojo Brillante in two maturity stages (S1 and S2). Vertical bars represent Least Significant Differences (LSD) intervals (*p* ≤ 0.05).

During persimmon drying, an increase in total soluble solids content occurred, mainly due to drop in moisture content as TSS are a major component of dry matter [33]. The TSS content at harvest was 15.9 °Brix for S1 and 14.7 °Brix for S2 for whole fruit. Throughout drying, TSS content gradually increased and its evolution was similar in both stages with values close to 47 (Figure 5A).

When the TSS content was measured separately in the outermost area and internal flesh, major differences appeared (Figure 5B). In the external area, a major increase continued until day 28 in both stages, from which time S1 continued to gradually increase and S2 tended to stabilize before slightly increasing again from day 56. After 63 days, TSS content reached mean values of 48.4 °Brix and 49.1 °Brix for S1 and S2, respectively. In flesh, TSS content gradually rose, but it was slower than in the external area, albeit with similar values close to 45 by the end of the experiment. The differences in the TSS content concentration between the two fruit parts are related to different moisture losses during drying. Thus, the higher values in the secondary epidermis throughout the experiment would indicate a higher proportion of TSS due to the more marked reduction in water in this fruit area.

Drying also decreases astringency in fruit due to insolubilization of tannins. This process is generally used to remove astringency from persimmon fruit in Asian countries. Changes in soluble tannin contents in astringent persimmon during drying have been established, which indicate a constant reduction in the level of tannins throughout the water loss process [34]. In this study, the astringency of variety “Rojo Brillante” was evidenced by a high soluble tannin content at harvest, with 4.5% DW in fruit at S1 and 2.6% DW (Figure 5C). These values agree with previous studies about “Rojo Brillante” [13,35].

S1 fruits showed a marked decrease in the first week, which was similar in both zones, while this decline was slower in S2 fruit (Figure 5D). When assessing internal and external zones separately, internal flesh on day 21 already had ST non-astringency values, which were close to 0.03% DW for both stages as shown by Tessmer et al. [35]. During the same period, astringency was still residual in the secondary epidermis, with values of 0.23% DW for S1 and 0.08% DW for S2. Fruit completely lost astringency only from day 28 for both stages and fruit parts.

Previous studies reported the factors that may lead to insolubilization of tannins in persimmon fruit. This process was mainly studied during fruit ripening. The softening that occurs during fruit maturation was related to the degradation of cell wall polysaccharides, which induce an osmotic pressure that causes the concentration of soluble tannins in the tannin cells, leading to its insolubilization [36]. Taira and Ono [37] suggested that the flesh cells collapsing during the ripening process leads to the production of cell wall fragments that adhere to the tannins, thus reducing astringency. Taira et al. [38] also reported that the softening and flesh cells collapse that occur during the fruit ripening lead to a solubilization of pectins which form a complex with tannins and causes their insolubilization. So, the tannins insolubilization during the drying process may also be associated to the structure changes in the fruit flesh. In this way, Asgar et al. [39] related the flesh softening that occurs during sun-drying of japanese persimmon to the solubilization and depolymerization of pectic polysaccharides.

### 3.5. Microstructural Changes

The microstructural study was carried out throughout persimmon drying by the Cryo-FESEM technique. For both maturity stages, from 7 days onward the structural evaluation was done for the two separate and differentiated areas: (1) internal flesh; (2) the secondary epidermis formed during the drying process.

#### 3.5.1. Internal Flesh

**Figure 6:**
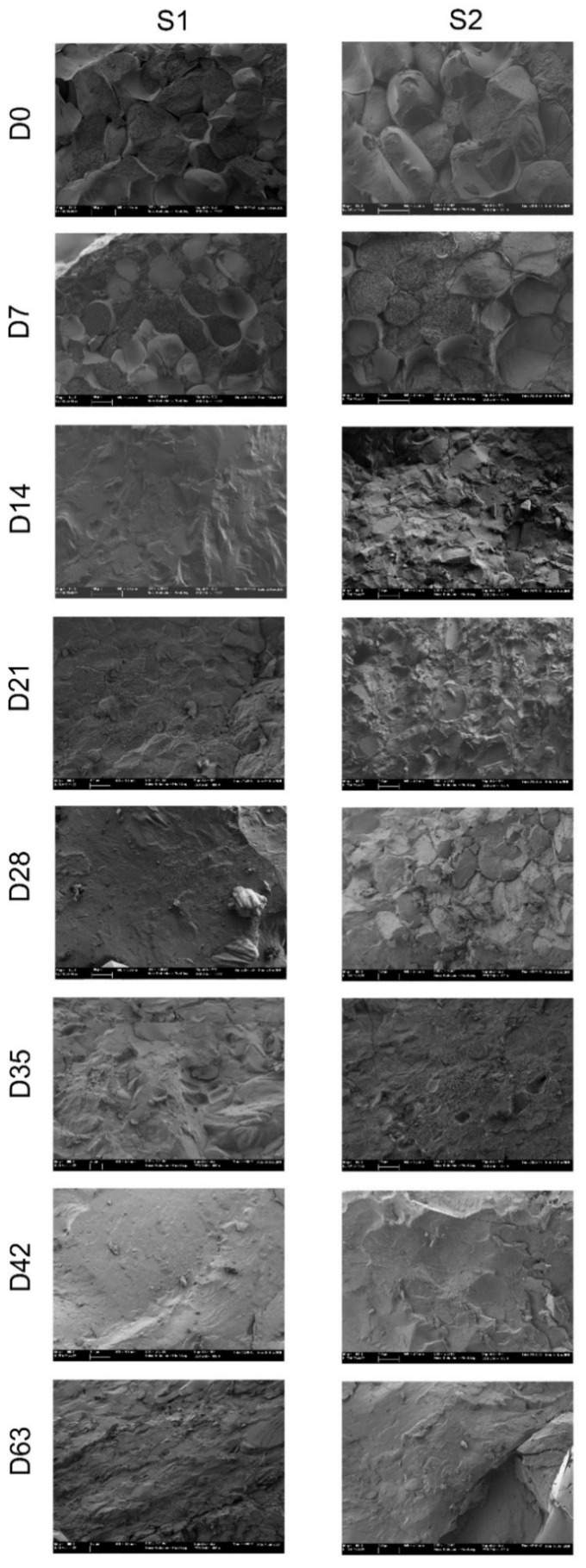
Internal flesh structure by the Cryo-field emission scanning electron microscopy technique of persimmon cv. Rojo Brillante in two maturity stages (S1 and S2) submitted to drying up to 63 days (D).

When Cryo-FESEM was used to observe a cross-section of persimmon internal flesh at harvest (day 0), the parenchyma was quite compact in both maturity stages (S1 and S2) and was formed by turgid and intact cells with intercellular spaces full of air and cell walls and the plasma membrane was intact (Figure 6).

During drying, cell structure deterioration became evident. In both stages, a drastic change in parenchyma structure was observed after 14 drying days onward. In S2, the parenchyma degradation process became slower while the drying process advanced in S1. On day 14 in S1, cell walls and cell membranes were hard to distinguish in the collapsed tissue. This cell structure deterioration continued as drying advanced. On day 28, the initial parenchyma structure had completely disappeared and had become a homogeneous compact mass in which no cells were visible. In S2, the cellular structure after 14 days had greatly deteriorated as most cells had lost their integrity and intercellular spaces were waterlogged, but unlike S1, it was still possible to see some structured cells. The total loss of the parenchyma structure with the appearance of a structure-less homogeneous mass occurred on day 42.

Parenchyma degradation is related to water removal, which occurs during drying. It is noteworthy that when the structure was completely lost and only a compact homogeneous mass was visible (on day 28 for S1, on day 42 for S2), fruit presented similar moisture content, which came close to 0.41 gH_2_O/g, and similar water activity, close to 0.87, when the measurements were taken on the whole fruit. When we observed the internal flesh data obtained, similar values were recorded for moisture, close to 0.56 gH_2_O/g, and water activity, close to 0.93, for S1 on day 28 and for S2 on day 42. After 63 days, the images of S1 showed more contracted tissue, but this was not found in S2.

In persimmon, the soluble tannins responsible for astringency are located inside vacuoles of the so-called tannic cells. In previous studies, the insolubilization of tannins during the ripening process has been observed by the Cryo-FESEM technique [13,34]. When tannins are soluble, they can be observed as a network, which is the typical eutectic artefact generated during the water sublimation process when samples are prepared by Cryo-FESEM. On the contrary, when tannins are insolubilized, a compact mass inside the vacuole is shown and no eutectic artefact can be seen. In the present study, cells with soluble material inside them were observed in the samples upon harvest. After day 7 in S1 and until day 14 in S2, although tissue was already degraded, some cells with soluble material were seen. Nevertheless, in the subsequent samples, no soluble material was observed, which might indicate that tannin insolubilization had occurred during the drying process. This corroborates the drastic drop in the content of soluble tannins to non-astringency values, which took place after 14 days or 21 days of drying in S1 and S2, respectively. This process would be similar to ripening since in the maturity process, persimmon has been reported to be accompanied by a gradual insolubilization of tannins [35]. Specifically, for the cultivar “Rojo Brillante”, complete astringency loss does not occur until fruit are overripe and their texture is very soft. In this state, pulp has a gel structure [35].

**Figure 7:**
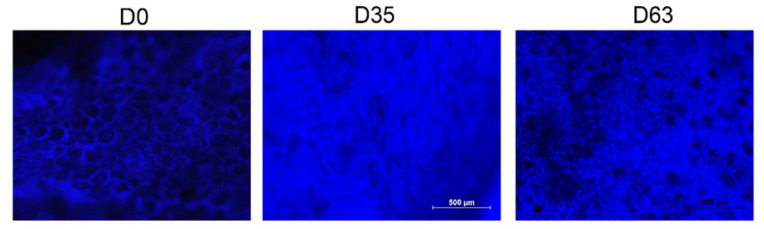
Tissue sections of persimmon cv. Rojo Brillante flesh submitted to drying observed by light microscopy (D means days of drying).

The gelling of flesh in the internal area was also observed under a light microscope by the fluorescence technique (Figure 7). Calcofluor was used as a staining agent, which is specific to cell walls (blue). The intact flesh upon harvest and the gelled tissue as a result of the drying process were compared. In the samples taken at harvest, cell walls were clearly visible. Cells are relatively spherical and uniformly distributed throughout the parenchyma. Nevertheless, in the samples of the gelled flesh, cell walls appeared torn and destroyed, which leads to the complete loss of structure tissue before collapse. The drastic degradation of cell walls would cause the leaching of cellular constituents into the tissue matrix. Tissue appears as tightly jumbled misshapen cells. At the end of the assay, after 63 days, tissue appears compacted as a result of the shrinkage process.

#### 3.5.2. Secondary Epidermis

**Figure 8:**
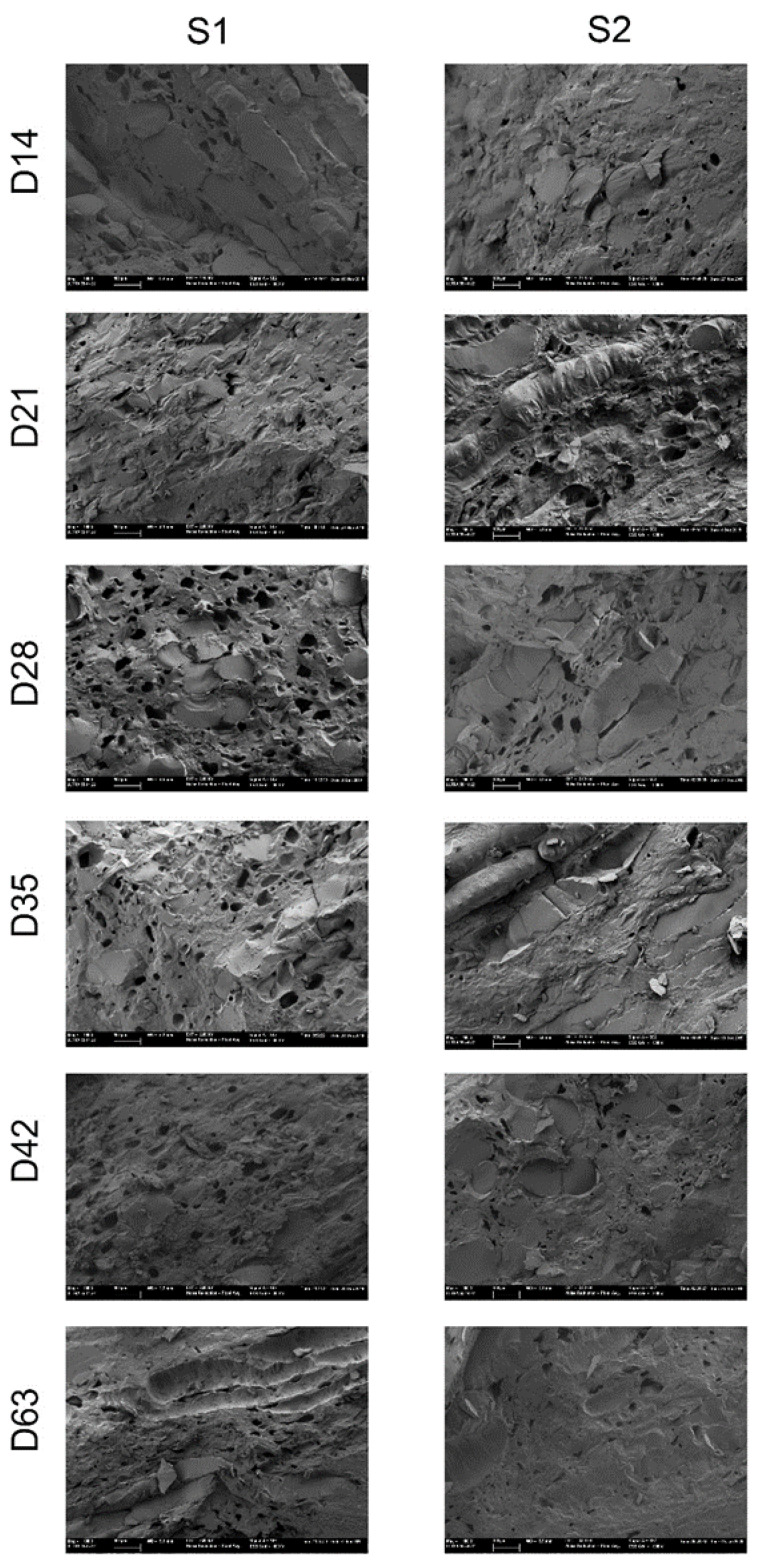
External flesh structure by the Cryo-field emission scanning electron microscopy technique of persimmon cv. Rojo Brillante in two maturity stages (S1 and S2) submitted to drying up to 63 days (D).

Figure 8 shows the microstructure of the secondary epidermis formed during drying process in both the maturity stages. The Cryo-FESEM evaluation was made from day 14 when the secondary epidermis was evident in both maturity stages.

After 14 days, the parenchyma of the fruit from both stages appeared to have collapsed and displayed a compact morphology. Cell walls were completely lost and the cell and intercellular spaces were full of insoluble material. Samples presented hollows, which were not observed in internal flesh. While some cells remained rounded in S2, the S1 tissue seemed more contracted and displayed squashed cell layers.

With the drying process, parenchymatic tissue evolves to become a more compact and destroyed structure due to the shrinkage process and leads to cell deformation. When observing internal flesh, the collapse of the structure in the secondary epidermis was faster in S1 than in S2.

The hollows displayed in these samples would be generated by the water vapor outflow during the drying process. The pore size in the samples from S1 was bigger than that from S2. The presence of pores in this structure could explain that internal flesh continues to lose water, even though this second epidermis had formed. Furthermore, the larger pore size in S1 would also explain the faster internal dehydration in S1 than in S2.

## 4. Conclusions

The physico-chemical and microstructure changes that take place during the drying of persimmon cv. Rojo Brillante in two maturity stages were studied. A characterization of the internal area (flesh) and external area (secondary epidermis formed during drying) of fruit was made for the first time. The obtained results revealed that maturity stage influences fruit characteristics during the drying process. Moisture loss was faster in S1 than in S2. Fruit had moisture values of semidried persimmon, i.e., around 0.5 gH_2_O/g, after 21 and 28 days for S1 and S2, respectively.

During the drying process, the formation of a secondary epidermis, concomitantly to internal flesh gelification, was related to the moisture loss that occurred in fruit in each maturity stage. This process was slower in S2 than in S1. The changes in the parequimatic structure of the outmost area observed by Cryo-FESEM were related to the hardening of this area as a result of increased moisture loss compared to internal flesh. In internal flesh, texture changed as a result of parenchyma degradation, which led to a structure that looked like a homogeneous mass, in which cell membranes were completely degraded. Internal flesh gelling was also clearly observed under alight microscope using calcofluor to stain the cell walls. The collapsed tissue presented hollows generated by water outflow, which would allow water to be removed from the internal flesh. S2 presented a thinner secondary epidermis and a bigger volume of gelled area inside, which would make them more elastic and softer compared to S1. Astringency loss took place due to the insolubilization of tannins during the water loss process.

The present study revealed that “Rojo Brillante” persimmon is a suitable astringent variety to be submitted to a drying process after taking into account that the final product characteristics depend on the maturity state upon harvest.
